# Breast cancer and neurofibromatosis type 1: a diagnostic challenge in patients with a high number of neurofibromas

**DOI:** 10.1186/s12885-015-1215-z

**Published:** 2015-03-26

**Authors:** André Vallejo Da Silva, Fabiana Resende Rodrigues, Mônica Pureza, Vania Gloria Silami Lopes, Karin Soares Cunha

**Affiliations:** 1 Breast Surgery Service, Hospital Universitário Antônio Pedro, Universidade Federal Fluminense, Niterói, Rio de Janeiro Brazil; 2 Pathology Service, Hospital Universitário Antônio Pedro, Universidade Federal Fluminense, Niterói, Rio de Janeiro Brazil; 3 Postgraduate Program in Pathology, School of Medicine, Universidade Federal Fluminense, Niterói, Rio de Janeiro Brazil

**Keywords:** Neurofibromatosis 1, Genetic diseases, Breast cancer, Neurofibroma, Secondary prevention

## Abstract

**Background:**

Neurofibromatosis 1 is one of the most common genetic diseases in humans, presenting with multiple neurofibromas and an increased risk of various benign and malignant tumors, including breast cancer.

**Case presentation:**

In this paper we report a case of a woman with neurofibromatosis 1 and the challenge associated with detecting an advanced breast cancer because of numerous skin neurofibromas, which were responsible for a substantial delay in cancer diagnosis. Literature concerning the association of neurofibromatosis 1 and breast cancer is reviewed and discussed.

**Conclusions:**

Best practice guidelines for breast cancer detection are not sufficient for the screening of neurofibromatosis 1 carriers. A more intensive clinical and imaging approach should be used if the same early detection rate as in non-neurofibromatosis 1 women is to be achieved.

## Background

Neurofibromatosis 1 (NF1) is one of the most common genetic diseases in humans, with a prevalence of one case in 3,000 births. The disease is caused by mutations in the *NF1* gene, which is considered a classical tumor suppressor [1]. NF1 is an autosomal dominant condition with complete penetrance but an extremely variable phenotype. Multiple neurofibromas, café-au-lait spots, “freckling” in the inguinal and axillary regions and Lisch nodules develop in most affected individuals [1]. Beyond the development of neurofibromas, which are benign peripheral nerve sheath tumors, NF1 patients have an increased risk of developing other benign and malignant neoplasms, including gliomas, malignant peripheral nerve sheath tumors (MPNSTs), juvenile chronic myelomonocytic leukemia, rhabdomyosarcoma, and pheochromocytoma [2-4]. NF1 is also a risk factor for the development of breast cancer [5-7].

We report a case of a 54-year-old woman with NF1 and the challenge involved in detecting an advanced breast cancer because of numerous skin neurofibromas. We also review the literature concerning the association between NF1 and breast cancer.

## Case presentation

A 54-year-old woman with a diagnosis of NF1 according to the National Institutes of Health criteria [8] was referred to the Breast Service of the Hospital Universitário Antônio Pedro of Universidade Federal Fluminense by the Oral Diagnosis Service from the same institution with the complaint of a “secretion from a mass in her left breast”. The patient reported that she delayed consulting a physician because she thought the mass was a manifestation of NF1. She was post-menopausal and had no family history of breast or ovarian cancer.

**Figure 1:**
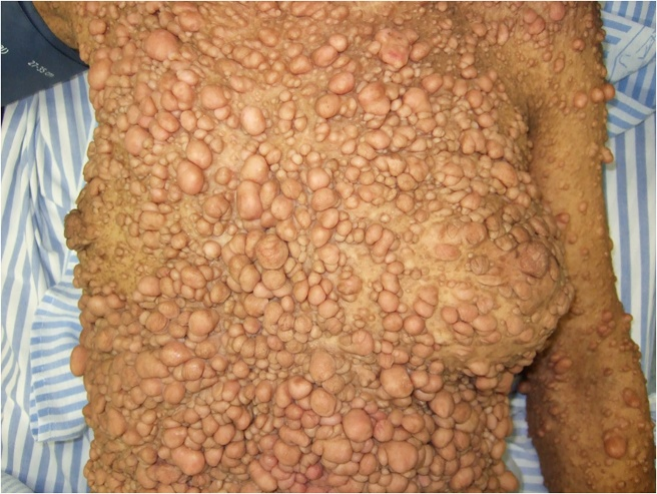
**Clinical aspect of the patient before mastectomy.** Photograph showing the high number of skin neurofibromas. Note that the left breast is enlarged.

**Figure 2:**
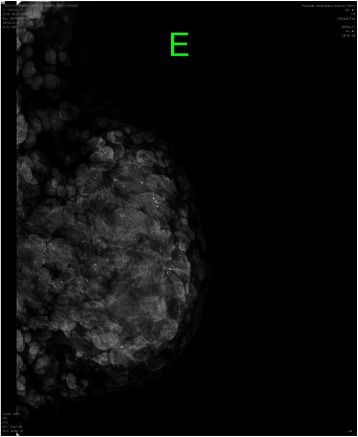
**Radiological aspect of the digital mammogram before surgery.** Mammogram illustrating the difficulty in identifying the tumor mass due to the high number of skin neurofibromas.

On physical examination, we observed thousands of neurofibromas all over her body, including both breasts (Figure 1). Left breast palpation revealed a large, tender mass occupying the whole breast of approximately 10 cm in diameter. Ipsilateral enlarged axillary lymph nodes were also palpated. Identification of the nipple-areolar complex was difficult because of the extension of her neurofibromas. At consultation, a needle core-biopsy was performed, and histopathological analysis revealed a grade 1 ductal carcinoma in situ. Digital mammograms were performed, but they were very difficult to interpret due to the extension of the cutaneous lesions. A large breast density associated with diffuse microcalcification was identified (Figure 2).

The clinical decision was to precede with a modified radical mastectomy and axillary clearance of levels I and II, because of the size of the mass. The surgical procedure was uneventful, with the skin incision contouring the neurofibromas. Of note, the skin flaps dissection showed some large vessels irrigating the skin, which necessitated great care when raising the flaps. Dissected axillary lymph nodes were large and soft. The healing process was normal, and the patient had a favorable evolution.

The histopathological analysis of the surgical specimen showed extensive high-grade ductal carcinoma in situ, comedo type, 10 cm in diameter and located in all breast quadrants, associated with an invasive ductal carcinoma measuring 0.4 cm. Mastectomy margins were free of disease. Histopathological analysis confirmed that the nodular cutaneous lesions were neurofibromas. Forty lymph nodes were isolated and showed only inflammatory reaction. Immunohistochemistry analyses revealed tumor cells negative for estrogen receptor (ER), progesterone receptor (PR), pan-cytokeratin (pan-CK), S100, vimentin and epithelial membrane antigen (EMA). Ki-67 was positive in 25-50% of tumor cells; Her2/neu was also positive (2+/3+). Fluorescence in situ hybridization (FISH) was inconclusive due to exhaustion of the invasive component of the tumor in the paraffin block. There was a small invasion of the pectoralis fascia in the upper inner quadrant, though no invasive tumor was found in the breast directly over it. Final pTNM staging was pT1N0M0.

The patient completed the chemotherapy treatment (cyclophosphamide, methotrexate and fluorouracil; six cycles), and is currently on a course of radiotherapy because of the size of the tumor. The medical oncologist decided on chemotherapy based on the size of the tumor mass, even with an invasive portion of less than one centimeter because of the aggressive biology of the tumor observed by immunohistochemistry and also because of the worse prognosis of breast cancer in NF1 patients reported in the literature.

## Discussion

Although digital mammography is the gold standard for screening for early stage breast cancer, in this paper we highlight the challenge in interpreting images of a large breast carcinoma in an NF1 patient due to the high number of skin neurofibromas. Physical examination of this patient was also somewhat impaired because of the amount of cutaneous lesions. The difficultly in detecting breast cancer in NF1 individuals with numerous skin neurofibromas has been reported previously [4,9,10]. NF1 can obscure or delay the identification of breast lesions not only because skin neurofibromas can mask the signs of a malignant lesion, but also because patients and physicians may mistakenly consider a breast mass to be a manifestation of the primary disease [11].

The first report of an association between NF1 and breast cancer was published in 1972 [12]. Several other clinical cases of NF1 patients with breast cancer were subsequently presented in the literature [4,9-11,13-17]. Because breast cancer is a common tumor in the general female population, the exact relationship between NF1 and breast cancer has been debated. To date, the study with the largest cohort of NF1 individuals (n = 448) that investigated the prevalence of breast cancer, as well as other types of cancer, showed that the risk of breast cancer was significantly higher in NF1 patients younger than 50 years of age than in the general population [5]. Sharif *et al.* [6] identified 14 cases of breast cancer within a cohort of 304 NF1 women older than 20 years, which represented a 3.5-fold risk of breast cancer in association with NF1. The same study calculated a 4.9-fold risk of developing breast cancer up to age of 50, representing an 8.4% cumulative risk of developing breast cancer compared with the risk in the general population of 2%. In a cohort of 126 patients, Madanikia *et al.* [7], in a retrospective study with 506 NF1 patients, identified four cases of breast cancer, and found a trend for an almost 3-fold increase in the risk of breast cancer in women with NF1 who were <50 years old compared with age-matched unadjusted incidence rates.

The data showing a high prevalence and a possible earlier onset of breast cancer in NF1 individuals have important implications on screening [5]. Breast cancer screening guidelines have been delineated for the general population and for women with known genetic risk factors for breast cancer (i.e. BRCA1 and PTEN syndromes) to decrease mortality through early diagnosis; however, there are currently no such guidelines for NF1 patients [7].

A study of over 1,000 NF1 individuals found an incidence of breast cancer mortality in NF1 females of approximately 3.5-fold that of the general population [18]. This finding suggests that not only is there an increased risk and earlier onset of breast cancer in women with NF1, but also a worse prognosis associated with the disease. As occurred in our patient, most of the cases of breast cancer in NF1 individuals are diagnosed at an advanced stage with a T score greater than 2 [9,13]. Therefore, the worse prognosis of breast cancer in NF1 may not be a characteristic of the disease itself, but may result from late-stage diagnosis due to the presence of skin neurofibromas, which hinder its identification, or due to the delay in seeking medical care by patients who think the breast mass is a neurofibroma. The most common histopathological type of breast cancer in NF1, as well as in the general population, is infiltrating ductal carcinoma [7], as observed in our case.

The scientific data support a real association between breast cancer and NF1. With regard to this, various authors have suggested different mechanisms supporting the relationship between NF1 and breast cancer. One of the implicated oncogenic events in breast cancer is the overexpression of Ras, which occurs in up to 60% of all cases and exerts several effects including perturbed cytoskeletal structure, decreased cell survival and increased apoptosis [19]. Neurofibromin, the protein product of *NF1* gene, which is located on chromosome 17q11.2, functions as a negative regulator of the Ras pathway, interacting with Ras and converting active Ras-GTP to its inactive form, Ras-GDP. The *NF1* gene acts in accordance with the Knudson two-hit hypothesis: in NF1-related tumors, biallelic inactivation of the *NF1* gene results in complete loss of functional neurofibromin activity [13]. In addition to upregulation of Ras, absence of neurofibromin expression has been observed in breast cell lines [20], suggesting overlapping etiologies [13]. However, it is not known whether the lack of neurofibromin is a primary or a secondary event in breast cancer tumorigenesis. Interestingly, around 30% of sporadic breast cancers in humans lack at least one copy of *NF1* gene [15].

Mutations of the tumor suppressor genes *BRCA1* and *BRCA2* are known to be associated with different patterns of hereditary breast and ovarian cancer [13]. Because *BRCA1*, like *NF1*, is located on human chromosome 17q, it has been suggested that an interaction could exist between these two genes [6]. While it is probable that some individuals reported in the literature carried mutations in both *BRCA1* and *NF1* genes, there is a scarcity of reports of germline mutations in both genes in the same individual. To the best of our knowledge, Campos *et al.* [13] were the only group to report a family with individuals with diagnosis of NF1 and breast cancer who were carriers of both *BRCA1* and *NF1* mutations. They concluded that the concurrence of NF1 and breast cancer was probably due to the simultaneous existence of two cancer-predisposing conditions.

## Conclusion

In conclusion, it is important that patients and physicians are aware of the increased risk of breast cancer in the NF1 subset. For early detection, it seems that the best practice guidelines used to screen women in the general population are not sufficient for NF1 patients. Published data justify earlier screening programs designed specifically for this group, including annual physical examination by a breast specialist. The problems presented by interpreting mammograms from NF1 patients highlight a need for more intensive non-mammographic breast imaging studies in patients with high numbers of neurofibromas. Examples of these include ultrasound and magnetic resonance imaging. The sensitivity of which are unlikely to be affected by cutaneous lesions, and they have the added advantage of an absence of radiation-related side effects. It is clear that more studies are necessary to clarify the relationship between these two diseases and to develop specific screening guidelines if earlier diagnosis and decreased morbidity and mortality are to be achieved in women with NF1 and breast cancer.

## Consent

Written informed consent was obtained from the patient for publication of this case report and the accompanying images. A copy of the written consent is available for review by the Editor-in-Chief of this journal.

## Abbreviation

NF1: Neurofibromatosis type 1
